# The Cornea: No Difference in the Wound Healing Response to Injury Related to Whether, or Not, There’s a Bowman’s Layer

**DOI:** 10.3390/biom13050771

**Published:** 2023-04-29

**Authors:** Steven E. Wilson

**Affiliations:** The Cole Eye Institute, I-32, Cleveland Clinic, 9500 Euclid Ave, Cleveland, OH 44195, USA; wilsons4@ccf.org; Tel.: +1-216-444-5887

**Keywords:** Bowman’s layer, corneal wound healing, fibrosis, myofibroblasts, keratocytes, corneal fibroblasts, TGF beta, epithelial basement membrane

## Abstract

Bowman’s layer is an acellular layer in the anterior stroma found in the corneas of humans, most other primates, chickens, and some other species. Many other species, however, including the rabbit, dog, wolf, cat, tiger, and lion, do not have a Bowman’s layer. Millions of humans who have had photorefractive keratectomy over the past thirty plus years have had Bowman’s layer removed by excimer laser ablation over their central cornea without apparent sequelae. A prior study showed that Bowman’s layer does not contribute significantly to mechanical stability within the cornea. Bowman’s layer does not have a barrier function, as many cytokines and growth factors, as well as other molecules, such as EBM component perlecan, pass bidirectionally through Bowman’s layer in normal corneal functions, and during the response to epithelial scrape injury. We hypothesized that Bowman’s layer represents a visible indicator of ongoing cytokine and growth factor-mediated interactions that occur between corneal epithelial cells (and corneal endothelial cells) and stromal keratocytes that maintain the normal corneal tissue organization via negative chemotactic and apoptotic effects of modulators produced by the epithelium on stromal keratocytes. Interleukin-1 alpha, produced constitutively by corneal epithelial cells and endothelial cells, is thought to be one of these cytokines. Bowman’s layer is destroyed in corneas with advanced Fuchs’ dystrophy or pseudophakic bullous keratopathy when the epithelium becomes edematous and dysfunctional, and fibrovascular tissue commonly develops beneath and/or within the epithelium in these corneas. Bowman’s-like layers have been noted to develop surrounding epithelial plugs within the stromal incisions years after radial keratotomy. Although there are species-related differences in corneal wound healing, and even between strains within a species, these differences are not related to the presence or absence of Bowman’s layer.

## 1. Introduction

**Figure 1:**
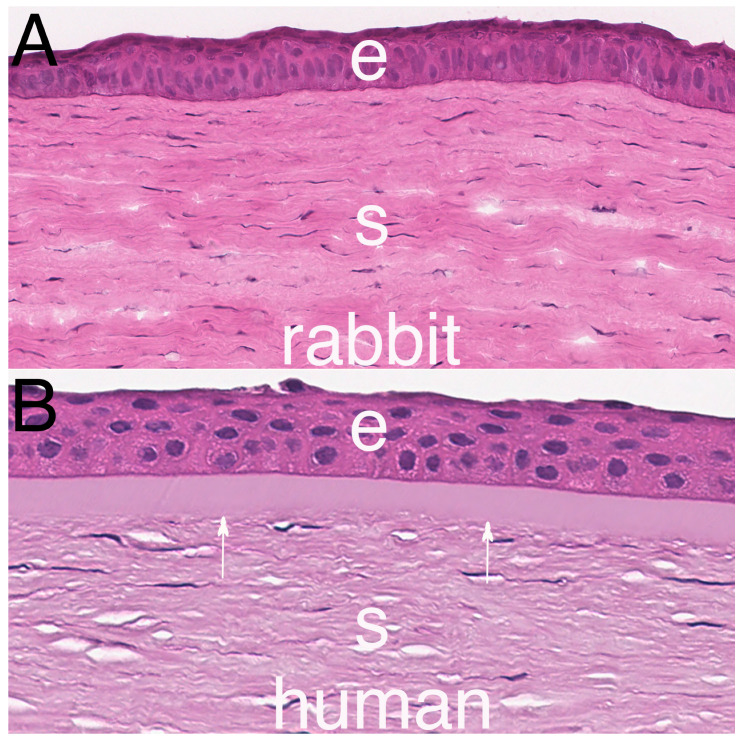
Hematoxylin and eosin staining of an anterior rabbit cornea (**A**) without Bowman’s layer and a human cornea (**B**) with Bowman’s layer (arrows). e is epithelium. s is stroma. Mag. 200×.

Bowman’s layer is an acellular layer of connective tissue that lies just posterior to the epithelial basement membrane (EBM) (Figure 1) in the species that have the layer. Bowman’s layer is found in the cornea of humans, most primates (other than lemurs), chickens, quails, zebra fish, deer, ox, giraffes, antelopes, California sea lions, zebus, elands, and guinea pigs [1,2,3,4,5,6,7,8,9,10]. It is not present in the corneas of dogs, wolves, cats, tigers, lions, pigs, cows, goats, horses, or rabbits [1,2,3,4,5,6,7,8,9,10]. There is controversy regarding Bowman’s layer in mouse corneas. One study [2] reported it is present in C3H and DDY mouse strains, but it was not found with transmission electron microscopy (TEM) in BALB/c and C57 BL/6 mice [11,12], with the latter publication providing TEM images from C57 Bl/6 mice. This could be related to strain variations in mice.

## 2. Bowman’s Layer Structure

Bowman’s layer is eight to eighteen µm thick in adult humans and is composed of randomly oriented collagen fibrils [13,14,15,16]. Bowman’s layer collagen fibrils are only 50% to 66% the diameter of fibrils in the deeper stroma [17]. It has been shown that the anchoring fibrils that extend between the basal epithelial cells and the anterior stroma were the same in humans with Bowman’s layer and rabbits without Bowman’s layer [16]. Rabbits, that do not have a Bowman’s layer in the adult, do have a Bowman’s-like layer early in development [18,19,20].

Similar to the underlying stroma, collagen type I, is the dominant collagen component in Bowman’s layer [2,10] but other collagens, including types III, V, and XII, are also present in these Bowman’s layer fibrils [3,4,17,21,22,23]. Gordon and coworkers [24] proposed that collagen type XII present in the Bowman’s layer fibrils contribute to the stability of these fibrils. Moreover, a higher concentration of the beta Ig-h3-coded protein keratoepithelin has been reported in Bowman’s layer compared to the more posterior stroma [1].

## 3. Bowman’s Layer Function

Any hypothesized functions for Bowman’s layer must account for the millions of patients who have had photorefractive keratectomy (PRK) in the world since 1988 and had Bowman’s layer removed from their central corneas with subsequent normal corneal function without apparent sequelae. Admittedly, rare patients develop complications after PRK, such as ectasia and scarring fibrosis despite mitomycin C treatment (breakthrough haze), but these represent only a tiny proportion of the total patients who have had PRK over the last thirty-plus years. This is not meant to minimize the complications that can develop in some patients after PRK, often because they were poorly selected prior to surgery, but they represent a rather small proportion of the total cases.

Tong et al. [25] proposed that Bowman’s layer had a mechanical role in maintaining corneal shape and this has led to surgical approaches to keratoconus in which cadaveric Bowman’s layer transplantation, often into the mid-stroma of the corneas with keratoconus, was suggested to treat and halt the progression of the disease [25,26,27]. However, Seiler and coworkers [28] found that Bowman’s layer did not contribute significantly to mechanical stability within the cornea. Moreover, studies of Bowman’s layer transplantation reported to date have not included controls in which deeper stromal tissue was used to fashion the lamellar transplant and, therefore, provide evidence that the Bowman’s tissue itself was critical in the treatment.

**Figure 2:**
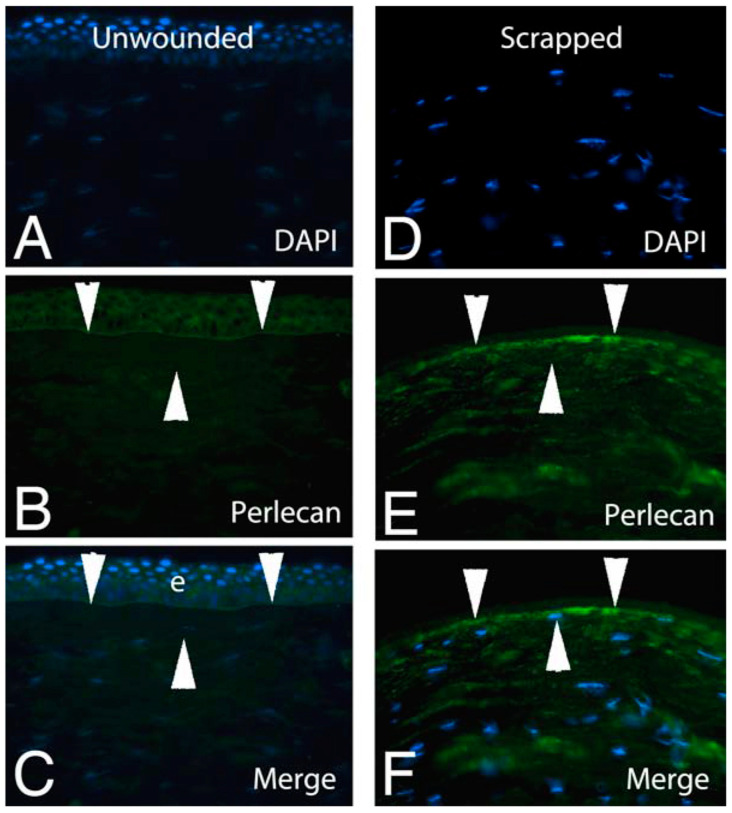
Immunohistochemistry for EBM component perlecan (470 kDa) in the normal unwounded human cornea (**A**–**C**) compared to a normal human cornea thirty minutes after epithelial scrape injury (**D**–**F**) prior to enucleation for choroidal melanoma. Perlecan produced by the stromal cells penetrates full-thickness Bowman’s layer (area between the two superior and one inferior arrowheads in each panel) either from keratocytes that have upregulated perlecan production (this can be seen throughout stroma in (**E**,**F**)) or from the scraped epithelial cells as they were removed, or possibly both, to accumulate at the site of nascent EBM regeneration. Blue is DAPI staining of cell nuclei in all panels. e is epithelium. Mag. 400×. Reprinted with permission from Ref. [41]. Copyright 2015 Experimental Eye Research.

The author attended conferences in which attendees suggested that Bowman’s layer provided some sort of barrier to the passage of growth factors and other modulators bidirectionally from the epithelium and the stroma. However, molecules, such as hepatocyte growth factor and fibroblast growth factor-7 (103 and 28 kDa, respectively), pass through Bowman’s layer to modulate stromal–epithelial interactions in which stromal cells regulate the proliferation, migration, and differentiation of corneal epithelial cells [28,29]. Similarly, if the corneal epithelium and EBM are gently scrapped, so that Bowman’s layer is not damaged, anterior stromal keratocytes undergo apoptosis that is regulated, at least in part, by epithelial interleukin-1 alpha (IL-1α, 18 kDa) released by the injury [11]. This stromal IL-1α binds IL-1 receptors on keratocytes to trigger autocrine suicide via the Fas-Fas ligand system [30,31]. Moreover, residual posterior and peripheral keratocytes undergo transformation to corneal fibroblasts driven by transforming growth factor (TGF) beta-1 (activated 25 kDa), TGF beta-2 (activated 25 kDa), and platelet-derived growth factor (PDGF) (25 kDa) that enter the deeper stroma from the tears and epithelium [32,33,34,35]. The critical barrier functions in the anterior cornea have been shown to be performed by the EBM and apical epithelial barrier function [32,33]. For example, the EBM components perlecan and collagen type IV are critical modulators of TGF beta-1 and TGF beta-2 entry into the stroma from the epithelium and tears, and defective regeneration of the EBM underlies the development of stromal fibrosis (haze) after corneal injury [32,33,36]. Moreover, after injury, the EBM has been shown to regenerate through the coordinated actions of epithelial cells and keratocytes/corneal fibroblasts [32,33,36]. In order for this coordination to occur in eyes with Bowman’s layer, large EBM components, such as perlecan produced by keratocytes and corneal fibroblasts, must pass through Bowman’s layer to the site of nascent EBM regeneration (Figure 2).

Are the wound healing responses different in corneas with and without Bowman’s layer? Studies examining important components of the corneal wound healing response after injury in chickens with Bowman’s layer, including keratocyte apoptosis, corneal fibroblast development, and myofibroblast development [37,38,39], have not found differences compared to these responses in corneas without Bowman’s layer, such as rabbits [32,33]. Studies of these responses in human corneas in situ using similar methods are necessarily limited but there are a few that can be cited. Human corneas that had epithelial scrape injury prior to enucleation for retinoblastoma were noted to have a similar anterior stromal keratocyte apoptosis response to rabbit and mouse corneas [40]. Moreover, perlecan and nidogen-2 proteins, two EBM components, are upregulated in stromal cells after epithelial scrape injury to human corneas [41], similar to what was found in rabbit corneas [42] and passes through Bowman’s layer after epithelial scrape injury [41].

This is not meant to infer that there are not species variations in different aspects of the wound healing responses to corneal injury. Clearly, there are differences. For example, in humans that have high-correction PRK (-9D, for example) without mitomycin C, the incidence of stromal scarring fibrosis haze mediated by myofibroblast development is around two to five percent [43]. However, when rabbits have the same treatment, the incidence of stromal scarring fibrosis is virtually 100% [44]. Conversely, mouse strains with corneas that do not have Bowman’s layer (C57BL/6) are relatively resistant to the development of myofibroblast-mediated stromal fibrosis after PRK equivalents, and investigators must use methods, such as irregular phototherapeutic keratectomy (PTK), over a fine mesh screen to impede EBM regeneration to trigger significant anterior stromal fibrosis in mice [45]. Even within different strains of mice, there are variations in the myofibroblast-mediated stromal fibrosis response to irregular PTK that is not related to Bowman’s layer [46].

So, what then is the significance of Bowman’s layer in species that have it? The author forwarded the hypothesis that Bowman’s layer represents a visible indicator of ongoing cytokine and growth factor-mediated interactions that occur between corneal epithelial cells (and corneal endothelial cells in the posterior cornea) and keratocytes that maintain the normal corneal tissue organization [12,47]. The processes that maintain Bowman’s layer likely involve negative-chemotactic and apoptotic effects of factors produced by the overlying epithelium on keratocytes [48,49]. The molecular factors that maintain corneal structure are poorly understood but are likely important during development and probably function to maintain tissue organization in the adult. These types of cellular interactions are likely important in maintaining tissue organization in all organs. It has been hypothesized that in the cornea IL-1α is one of the cytokines produced by the epithelium (and corneal endothelium) that is part of this organ structure maintaining system [11].

**Figure 3:**
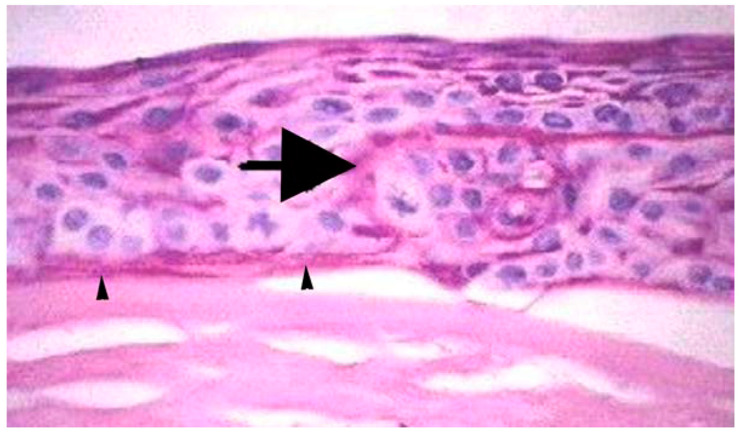
Bowman’s layer pathology. Bowman’s layer is destroyed in advanced bullous keratopathy when the epithelium becomes dysfunctional due to edema. In this cornea, ectopic fibrous tissue developed beneath the epithelium (arrowheads) and extended into the epithelium (large arrow). Hematoxylin and eosin staining. Mag. 200×. Reprinted with permission from Ref. [12]. Copyright 2020 Experimental Eye Research.

The support for this hypothesis is provided by diseases, such as Fuchs’ dystrophy or pseudophakic bullous keratopathy. As these disorders advance, the epithelium becomes edematous and dysfunctional as the corneal endothelial compromise progresses, Bowman’s layer is destroyed (Figure 3), and a fibrovascular pannus commonly develops between the epithelium and the edematous stroma [12,47,48].

Bowman’s layer does not appear to regenerate in humans after procedures, such as PRK. However, Bowman’s-like acellular zones have been described to develop after some types of corneal injuries. For example, in radial keratotomy surgery, nearly full-thickness radial incisions are made deep into the corneal stroma. “Epithelial plugs” often extend into these incisions during healing and may remain indefinitely within the stromal incisions after surgery. Melles and coworkers [49] showed that a “Bowman’s-like layer” may develop in the stroma surrounding the epithelial plugs when the corneas were evaluated histopathologically five or more years after radial keratotomy surgery. A Bowman’s-like layer was noted beneath the EBM ten years after PRK in a human cornea rejected for transplantation due to a history of prior surgery (Wilson SE, unpublished data, 2002). A Bowman’s-like layer did not yet develop around epithelial plugs in rabbits without Bowman’s layer at only one month after deep incisional injuries into the corneal stroma [50].

## 4. Conclusions

Bowman’s layer is present in the corneas of humans, most primates (other than lemurs), chickens, quails, zebra fish, deer, ox, giraffes, antelopes, California sea lions, zebus, elands, and guinea pigs but is not present in the corneas of the dogs, wolves, cats, tigers, lions, pigs, cows, goats, horses, or rabbits. There may be strain variations in the presence of Bowman’s layer in mice. Bowman’s layer likely does not contribute significantly to the mechanical stability of the cornea. Bowman’s layer may be a visible indicator of ongoing stromal–epithelial interactions involved in the maintenance of corneal tissue organization and can be lost with the progression of diseases, such as Fuchs’ dystrophy and pseudophakic bullous keratopathy, when the epithelium becomes dysfunctional with the edema. Further studies are needed to explore the systems and factors that maintain corneal tissue organization in the adult human. Hypothesized roles for Bowman’s layer in corneas that have one remain controversial.

## Data Availability

Upon request.
